# Heme Oxygenase-1 Delays Gibberellin-Induced Programmed Cell Death of Rice Aleurone Layers Subjected to Drought Stress by Interacting with Nitric Oxide

**DOI:** 10.3389/fpls.2015.01267

**Published:** 2016-01-19

**Authors:** Huangming Wu, Yan Zheng, Jing Liu, Heting Zhang, Huiping Chen

**Affiliations:** College of Horticulture and Landscape Architecture, Hainan UniversityHaikou, China

**Keywords:** heme oxygenase-1, drought stress, *Oryza sativa*, aleurone layers, programmed cell death, nitric oxide, gibberellin

## Abstract

Cereal aleurone layers undergo a gibberellin (GA)-regulated process of programmed cell death (PCD) following germination. Heme oxygenase-1 (HO-1) is known as a rate-liming enzyme in the degradation of heme to biliverdin IXα, carbon monoxide (CO), and free iron ions (Fe^2+^). It is a critical component in plant development and adaptation to environment stresses. Our previous studies confirmed that HO-1 inducer hematin (Ht) promotes the germination of rice seeds in drought (20% polyethylene glycol-6000, PEG) conditions, but the corresponding effects of HO-1 on the alleviation of germination-triggered PCD in GA-treated rice aleurone layers remain unknown. The present study has determined that GA co-treated with PEG results in lower *HO-1* transcript levels and HO activity, which in turn results in the development of vacuoles in aleurone cells, followed by PCD. The pharmacology approach illustrated that up- or down-regulated *HO-1* gene expression and HO activity delayed or accelerated GA-induced PCD. Furthermore, the application of the HO-1 inducer Ht and nitric oxide (NO) donor sodium nitroprusside (SNP) not only activated *HO-1* gene expression, HO activity, and endogenous NO content, but also blocked GA-induced rapid vacuolation and accelerated aleurone layers PCD under drought stress. However, both HO-1 inhibitor zinc protoporphyrin IX (ZnPPIX) and NO scavenger 2-(4-carboxyphenyl0-4, 4,5,5-tetramethylimidazoline-l-oxyl-3-oxide potassium salt (cPTIO) reserved the effects of Ht and SNP on rice aleurone layer PCD under drought stress by down-regulating endogenous HO-1 and NO, respectively. The inducible effects of Ht and SNP on *HO-1* gene expression, HO activity, and NO content were blocked by cPTIO. Together, these results clearly suggest that HO-1 is involved in the alleviation of GA-induced PCD of drought-triggered rice aleurone layers by associating with NO.

## Introduction

Programmed cell death (PCD) plays a critical role in regulating plant development and in protecting from stress. Cereal aleurone layers are an ideal model for studying the effects of different factors on PCD. The aleurone layers, which surround the starchy endosperm of cereal grains, synthesize, and secrete various enzymes to decompose stored nutrients of endosperm, which in turn are utilized for embryo growth during seed germination (Mundy and Rogers, 1986). After completion of secretion, aleurone layer cells undergo PCD, which is regulated by gibberellin (GA) and abscisic acid (ABA; Bethke et al., 1999). Hence, the PCD of aleurone layers is the central event controlling germination; it decides the germination of seeds and supports the early seedlings growth. Previous investigations have declared that the typical morphological features of PCD occurring in the dying cereal aleurone cells include the fusion of small protein storage vacuoles (PSVs) into bigger ones, which eventually result in a single, large, spherical vacuole occupying almost the entire cytoplasmic area (Bethke et al., 1999). This process of developing vacuoles is referred to an vacuolation (Domínguez et al., 2004). In addition, GA not only triggers the metabolic activation of dormant aleurone cells, but also accelerates its PCD (Fath et al., 2000). The reduced ability of GA-treated cells to scavenge reactive oxygen species (ROS) results in the PCD of aleurone layers (Fath et al., 2001). However, to date, studies on the features of aleurone layer cells in the germinating cereal seeds during PCD have mainly focused on barley, whereas those of other cereals including rice have not been investigated.

Nitric oxide (NO) is a downstream signaling factor that is largely involved in auxin-mediated events (López-Bucio et al., 2006), such as promoting lateral root and adventitious root growth (Pagnussat et al., 2002). In addition, NO not only participates in a hypersensitive response (HR), but also prevents cell death (Delledonne et al., 1998; Beligni et al., 2002). In GA-treated barley aleurone layers, NO delays the loss of catalase (CAT) and superoxide dismutase (SOD), thereby, postponing the occurrence of PCD (Beligni et al., 2002). More recently, NO has been shown to alleviate salt-induced oxidative stress in wheat seedlings (Xie et al., 2008), promote seed germination, and growth in collaboration with heme oxygenase-1 (HO-1) under osmotic stress (Liu et al., 2010).

Heme oxygenase (EC 1.14.99.3) is an intracellular enzyme that catalyzes the oxidative degradation of heme into biliverdin IXα (BV), free iron ions (Fe^2+^), and carbon monoxide (CO; Muramoto et al., 2002; Devadas and Dhawan, 2006; Gohya et al., 2006; Bilban et al., 2008). All three products also have important physiological functions. Biliverdin is subsequently reduced into bilirubin (BR) under the action of biliverdin reductase (Wilks, 2002), and bilirubin is a kind of strong endogenous antioxidant cytoprotectant (Baraňano et al., 2002; Jansen et al., 2010). In recent years, research has suggested that HO-1 possesses very strong antioxidant properties in different resistance to oxidative stress for his two by-products, namely, BV and BR (Shekhawat and Verma, 2010). Fe^2+^, as one of the products catalyzed by HO-1, induces the ferritin to increase the antioxidant properties of cells (Baraňano et al., 2000). Increasing evidence has confirmed that the effects of HO-1 on plant growth and development mainly depend on the gas signal molecule, CO. CO exerts its physiological function through the cGMP pathway (Xuan et al., 2007). HO and the HO/CO system, which are considered as frontline factors that combat oxidative damage, also play important roles in wheat root growth (Liu et al., 2010).

To date, three types of HO isozymes have been identified. HO-1 is inducible, whereas, HO-2 and HO-3 are constitutively expressed and have very low activity (Ryter et al., 2002, 2006; Kim et al., 2006). Only HO-1 can be induced by a variety of stimuli that provoke oxidative stress such as heme, heme derivatives (hemin and Ht), and non-heme inducers, including heavy metals, salt, H_2_O_2_, NO, and its releasing compounds. Exposure of rice seeds to 20% PEG, an HO-1 inducer, results in retardation of seed germination inhibition (data not shown). Therefore, HO-1 has been a research of interest because it exerts a variety of beneficial physiological functions in plants. The protection of HO-1 against oxidative damage has been demonstrated in wheat (Huang et al., 2006; Xie et al., 2008; Wu et al., 2011), soybean (Noriega et al., 2004; Yannarelli et al., 2006), *Arabidopsis* (Xie et al., 2011, 2012), and alfalfa (Han et al., 2008). Furthermore, HO-1 also participates in developmental processes in plants such as root formation (Xuan et al., 2008; Cao et al., 2011; Lin et al., 2012) and seed germination (Xu et al., 2011). The effect of auxin on adventitious root formation in cucumber is realized by rapidly activating the activity of HO, which in turn results in the HO product, CO, thereby triggering signal transduction events (Xuan et al., 2008). Enhancement of *HO-1* transcription and HO activities in the antioxidant defense system in soybean leaves subjected to lower levels of cadmium (Cd) stress (Noriega et al., 2004, 2007) or UV-B irradiation (Yannarelli et al., 2006). There is mounting evidence that shows that HO-1 functions by coordinately interacting with NO. Xuan et al. (2012) previously reported that NO located in downstream of the HO-1 inducer hemin promotes cucumber adventitious rooting, whereas Noriega et al. (2007) suggested that NO up-regulated HO-1 transcript levels and enhanced HO activities in soybean leaves.

The role of HO-1 in regulating PCD by GA, NO, and H_2_O_2_ has been elucidated in wheat (Wu et al., 2011, 2014). However, the role of HO-1 in regulating PCD, particularly the morphological changes involving vacuoles has not been investigated in rice aleurone layers. Moreover, a relationship between HO-1 and NO signaling has not been established. Based on these statements and considering that PCD is a key event of cereal seed germination, we investigated the relationship of HO-1, NO, and GA during PCD in rice aleurone layers subjected to drought stress. The aims of the present study were to elucidate the function of HO-1 in the alleviation of GA-induced PCD in rice aleurone layers and to confirm whether this effect is caused by an interaction with NO.

## Materials and Methods

### Chemicals

All chemicals were obtained from Sigma (St. Louis, MO, USA) unless stated otherwise. In the present study, 20% polyethylene glycol-6000 (PEG) was used to mimic drought stress. Ht, a HO-1 inducer, was used at a concentration of 1 μM (dissolved in 0.1 mM NaOH). Zinc protoporphyrin IX (ZnPPIX), an inhibitor of HO-1 (Fu et al., 2011; Xie et al., 2011; Bai et al., 2012; Cui et al., 2012), was used at a concentration of 10 μM (dissolved in 0.1 mM NaOH). 2-(4-carboxyphenyl)-4, 4,5,5-tetramethylimidazoline-1-oxyl-3-oxide potassium salt (cPTIO), as a specific NO scavenger, was used at a concentration of 200 μM. NO-specific fluorophore 4,5-diaminofluorescein diacetate (DAF-2DA) was purchased from Calbiochem (San Diego, CA, USA) and used at a concentration of 10 μM (dissolved in 0.01 mM DMSO). Gibberellic acid (GA) was used at a concentration of 50 μM (dissolved in alcohol). Sodium nitroprusside (SNP), a NO donor, was used at a concentration of 200 μM.

### Plant Material, Growth Conditions, and Treatments

Seeds of rice (*Oryza sativa* L. cv. You II 128) were sterilized with 0.1% potassium permanganate for 5 min and extensively washed with distilled water. To promote seed germination, the seeds were placed in a Petri dish containing two layers of filter paper that were moistened with sterile water at a constant temperature 25°C for 1 day. The embryos and the ends of the seeds were removed, and then transferred to Petri dishes containing two sheets of filter paper moistened with distilled water for 2 days. Then, under sterile conditions, the aleurone layers were stripped from de-embryonated half-seeds. Isolated aleurone layers were directly incubated in a medium containing 20% PEG alone, or in the absence or presence of 50 μM GA, 1 μM Ht, 200 μM SNP, 10 μM ZnPPIX, and 200 μM cPTIO, at various time points. Layers incubated in water alone were used as control. Each treatment was repeated at least three times, and 30 aleurone layers were used in each replicate.

### Quantitative Real-Time PCR (QRT-PCR) Analysis

Total RNA was isolated from 30 pieces of aleurone layers by using TRIzol Reagent (Invitrogen, Carlsbad, CA, USA). After being isolated, the RNA samples were treated with RNAase-free DNase (TaKaRa, Dalian, China) to eliminate traces of DNA, then the RNA was quantified by using a UV-1800 spectrophotometer (Shimadzu, Japan). Reverse transcription was performed using the PrimeScript RT reagent Kit (TaKaRa, Dalian, China), according to the manufacturer’s instructions. DNA-free total RNA (0.5 μg) from different treatment was used for first-strand cDNA synthesis in a 20-μL reaction volume containing 4 μL 5x PrimeScript Buffer 2, 1 μL PrimeScript RT Enzyme Mix, and 1 μL RT Primer Mix. The relative *HO-1* mRNA levels of the rice aleurone layers were quantified by QRT-PCR, using the SYBR Green real-time PCR Master Mix (TianGen, Beijing, China), following the manufacture’s procedures. The PCR Master Mix per reaction contained 9 μL of 20x SYBR Green (containing 2.5x Real Master Mix), 4 μL of cDNA, and 0.5 μL of each oligonucleotide primer. The analysis of real-time PCR data was based on the comparative threshold cycle (C_T_) method, which involved normalizing against the transcript levels of EF-1α. The sequences of the primers were used for PCR amplification were based on the following: *HO-1* gene (GenBank Accession Number: CA753857) upstream primer 5′-TCAAGGAACAGGGTCACACAA-3′, 5′-CCTCCAGCCGTATGAGCAA-3′ (downstream primer 5 amplified fragment 142 bp): reference gene of EF-1α (GenBank Accession Number: AA753281) upstream primer 5′-ACGGCAAAACGACCAAGAAG-3′, 5′-CAAGAACGGTGATGTGGTATGG-3′ (downstream primer 5 amplified fragment).

### Determination of HO Activity

For extraction of HO, 30 pieces of aleurone layers were homogenized in 15 mL of 25 mM HEPES-Tris cold buffer solution (pH 7.4) containing 250 mM mannitol, 3 mM EDTA, 3 mM EGTA, 250 mM KI, 1 mM DTT, 0.1 g⋅L^-1^ BSA, 10 g⋅L^-1^ polyvinylpyrrolidone (PVP), and 10% glycerol. The homogenates were filtered across four layers of gauze, and the filtrate was centrifuged at 60,000 ×*g* for 30 min at 4°C, then the supernatant was used for the determination of HO activity as described elsewhere (Han et al., 2008). The concentration of BV was evaluated using a molar absorption coefficient in 6.25 mM^-1^⋅cm^-1^ in 0.1 M HEPES-NaOH buffer (pH 7.2) and at a wavelength of 650 nm. One unit of activity (U) was determined by determining the quantity of the enzyme to produce 1 nmol of BV per 30 min. Determination of protein content was determined using the Coomassie brilliant blue method (Bradford, 1976), with bovine serum albumin as standard.

### Determination of Cell Viability and Death

Double fluorescence probes FDA and FM4-64 were used to determinate the viability of rice aleurone layers cells (Bethke and Jones, 2001). The layers were stained with 2 μg⋅mL^-1^ FDA (20 mM CaCl_2_) for 15 min, followed by 20 mM CaCl_2_ to remove background fluorescence, stained with 1 μg⋅mL^-1^ FM4-64 (20 mM CaCl_2_) for 3 min, then with 20 mM CaCl_2_ to wash away background fluorescence. Images of the aleurone layers were captured with a laser scanning confocal microscope (LSCM, FV1000, Olympus). Four fields of each aleurone layer were randomly selected, and at least three different aleurone layers were measured per treatment. The number of live and dead cells was counted to determine the percentage of viable cells in different fields, and the numbers were averaged for each half-seed.

### Detection of NO Content

The aleurone layers were treated with 0.1 M Tris-HCl buffer (pH 7.4) for 30 min, and then incubated with DAF-2DA (10 μM in 0.1M Tris-HCl buffer) in the dark for 2 h. Excess probes were removed by washing with 0.1 M Tris-HCl buffer three times, each for 15 min (Piantadosi, 2002). The distribution and imaging of NO molecules in the aleurone layers were scanned using a LSCM (FV1000, Olympus) at a moderate speed at an excitation wavelength of 488 nm and an emission wavelength of 500–530 nm.

### Statistical Analysis

Data were expressed as the mean ± standard error of at least three independent experiments, and statistical significance was estimated using Duncan’s multiple test (*P* < 0.05).

## Results

### HO-1 Inducer and NO Donor Up-Regulated *HO-1* mRNA and HO Activity of Rice Aleurone Layers Subjected to Drought Stress

**FIGURE 1:**
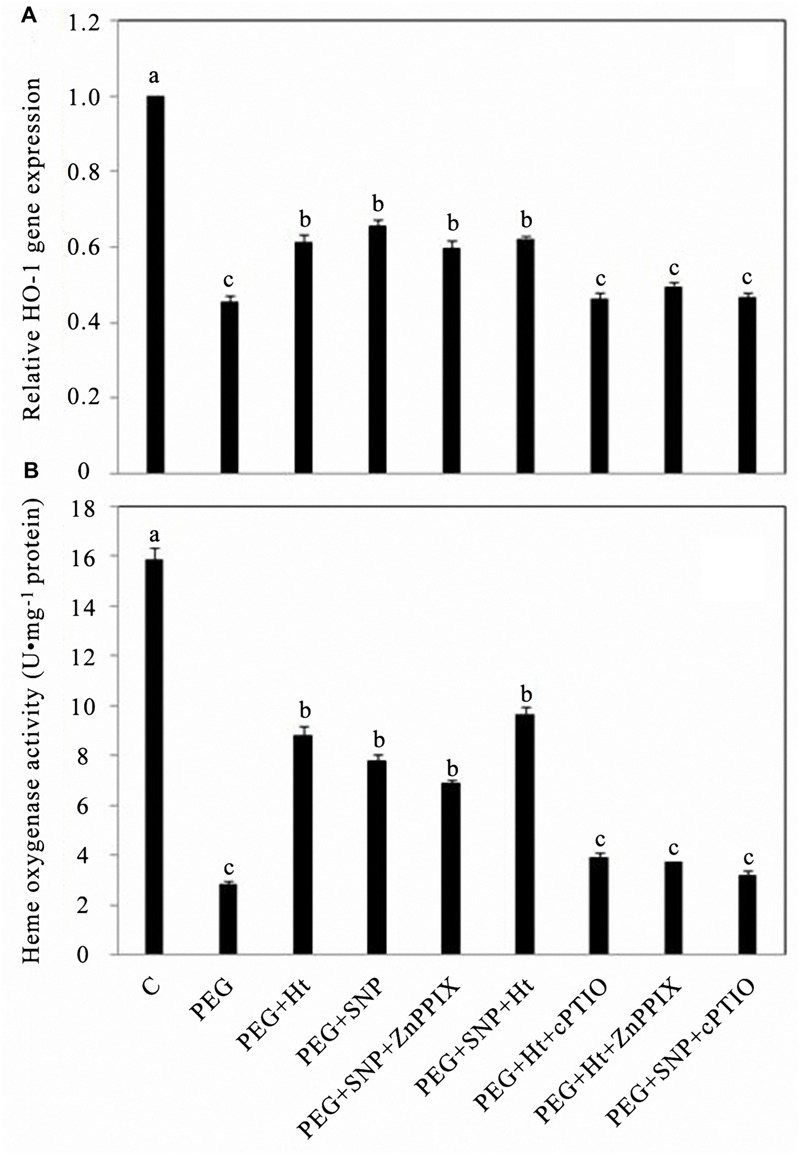
**Hematin (Ht), sodium nitroprusside (SNP), zinc protoporphyrin IX (ZnPPIX), and 2-(4-carboxyphenyl0-4, 4, 5, 5-tetramethylimidazoline-l-oxyl-3-oxide potassium salt (cPTIO) up- or down-regulated *HO-1* gene expression **(A)** and HO activity **(B)** in rice aleurone layers subjected to drought stress.** Rice aleurone layers were treated with or without 20% (polyethylene glycol-6000) PEG, 1 μM Ht, 200 μM SNP, 10 μM ZnPPIX, 200 μM cPTIO individually or in combination for 24 h. A sample with distilled water was used as control (C). Measurement of *HO-1* transcript levels in the rice aleurone layers was conducted by real-time fluorescence quantitative PCR assay **(A)**, HO activities were also detected **(B)**. Mean values were calculated from at least three independent experiments, bars with different letters indicate statistically significant differences at the 0.05 level, using Duncan’s multiple test.

To assess whether the different levels of HO activity caused by changes in *HO-1* mRNA expression, QRT-PCR was performed. PEG treatment alone significantly inhibited the transcription level of the *HO-1* gene in rice aleurone layers, whereas PEG co-treated with Ht and SNP led to the attenuation of PEG-induced *HO-1* gene expression. The combined treatment of PEG + Ht showed similar results as that observed using PEG + SNP. Compared to the treatments PEG + Ht and PEG + SNP, the PEG + SNP + Ht treatment did not significantly improve the expression of the *HO-1* gene, and no additive effect was observed using Ht and SNP. Similar results were observed in a study on de-etiolation of wheat seedling leaves (Liu et al., 2013). The effects induced by the HO-1 inducer, Ht, and the NO donor, SNP, were, respectively, reversed by ZnPPIX, a specific inhibitor of HO-1, and cPTIO, a scavenger of NO (**Figure 1A**).

Treatments including the NO donor, SNP, the HO-1 inhibitor, ZnPPIX, and PEG attenuated the decrease in HO-1 activity in aleurone layers treated with PEG alone (**Figure 1B**). Treatment using PEG + SNP and PEG + SNP + ZnPPIX for 24 h did not result in any distinct differences in the HO-1 activities of the aleurone layers, whereas these, respectively, increased by 1.95- and 2.58-fold compared to that using PEG alone (**Figure 1B**), which indicates that the HO-1 inhibitor, ZnPPIX, did not prevent the NO effect. Meanwhile, the observed changes in HO-1 activity were in agreement with the detected *HO-1* expression profile.

After treatment with PEG + Ht + cPTIO for 24 h, the relative expression level of *HO-1* and the activity of HO-1 in rice aleurone layers, respectively, decreased by 33.30 and 55.94% relative to that using PEG + Ht (**Figures 1A,B**). Meanwhile, no statistically significant differences between the treatments PEG and PEG + Ht + ZnPPIX were observed. These results indicated that the NO-specific scavenger cPTIO blocked the Ht-induced expression of *HO-1* by eliminating endogenous NO under drought stress. These results thus suggest that HO-1 acts downstream of NO in the rice aleurone layers under drought stress. Xuan et al. (2012) previously showed that HO/CO possibly acts as the downstream molecule of NO during the process of adventitious rooting in cucumber.

### HO-1 Increases the Level of Endogenous NO in Rice Aleurone Cells Under Drought Stress

**FIGURE 2:**
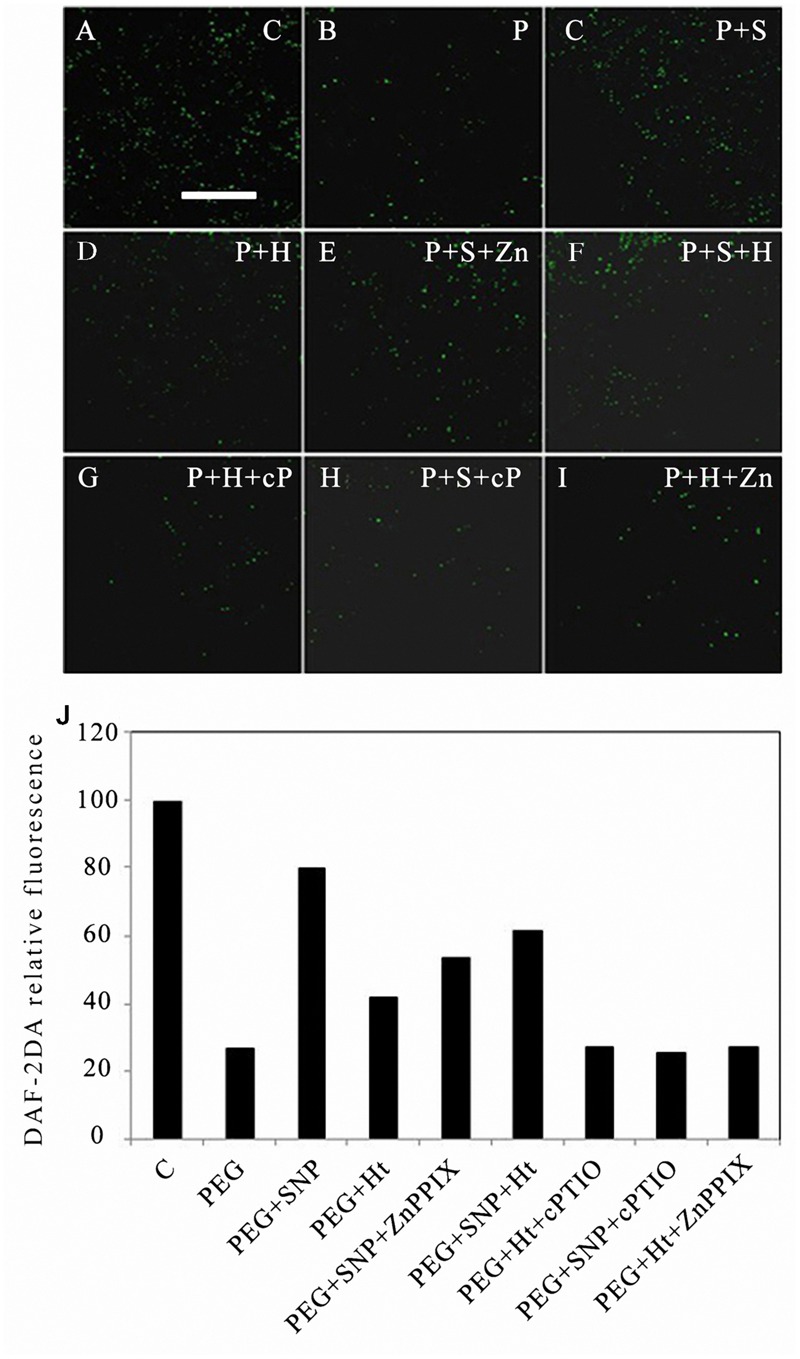
**Ht, SNP, ZnPPIX, and cPTIO raised or reduced endogenous NO content in rice aleurone layers under drought stress.** Rice aleurone layers were treated with or without 20% PEG (P), 1 μM Ht (H), 200 μM SNP (S), 10 μM ZnPPIX (Zn), and 200 μM cPTIO (cP) individually or in combination for 24 h. A sample with distilled water was used as control (C). After various treatments, the aleurone layers were, respectively, stained with DAF-2DA, and then thoroughly washed to removal excess dye and immediately observed under a LSCM. Images of the distribution of NO in fluorescently labeled aleurone cells were captured **(A–I)**. The relative DAF-2DA fluorescence intensity in the corresponding aleurone layers was also established **(J)**. Scale bar, 100 μm. Mean values were calculated from at least three independent experiments, bars with different letters indicate statistically significant differences at the 0.05 level, according to Duncan’s multiple test.

To further elucidate the relationship of upstream and downstream between HO-1 and NO during the PCD of rice aleurone layers subjected to drought stress, DAF-2DA, the specific NO fluorescence probe, was used to label the cells of the cultured rice aleurone layers subjected to various treatments for 24 h. The NO signal of cells was captured by LSCM (**Figures 2A–J**). The NO fluorescent signal was strong in rice aleurone cells under normal cultivation conditions, which clearly indicated that the level of intracellular NO was high (**Figure 2A**). In contrast, barely detectable NO fluorescent signals were observed under drought stress (**Figure 2B**), indicating that the formation of endogenous NO was significantly restrained under drought stress. However, the combined treatments of PEG + Ht (**Figure 2C**) or PEG + SNP (**Figure 2D**) showed stronger fluorescence signals compared to that using PEG alone, indicating that the HO inducer, Ht, induced NO synthesis in the rice aleurone cells under drought stress.

The combination of the NO donor, SNP, with the HO-1 inhibitor, ZnPPIX, effectively alleviated the decrease in the level of endogenous NO under drought stress (**Figure 2E**); therefore, the intracellular fluorescence intensity was distinct within 24 h of treatment, and was similar to that observed with PEG + SNP. These results indicated that the HO-1 inhibitor, ZnPPIX, did not reduce the synthesis of NO, which was induced by the NO donor, SNP, under drought stress. PEG + SNP + Ht (**Figure 2F**) treatments including the NO scavenger such as PEG + Ht + cPTIO and PEG + SNP + cPTIO effectively prevented NO formation, which was elicited by the HO-1 inducer, Ht, and the NO donor, SNP, in the rice aleurone layers after 24 h of treatment (**Figures 2G,H**). These findings indicated that the NO-specific scavenger, cPTIO, blocked the forming of intracellular NO, which was induced by HO-1 and SNP in the aleurone layers.

### GA-Induces the Down-Regulation of *HO-1* Gene Expression and Activity Level in Rice Aleurone Layers Subjected to Drought Stress

**FIGURE 3:**
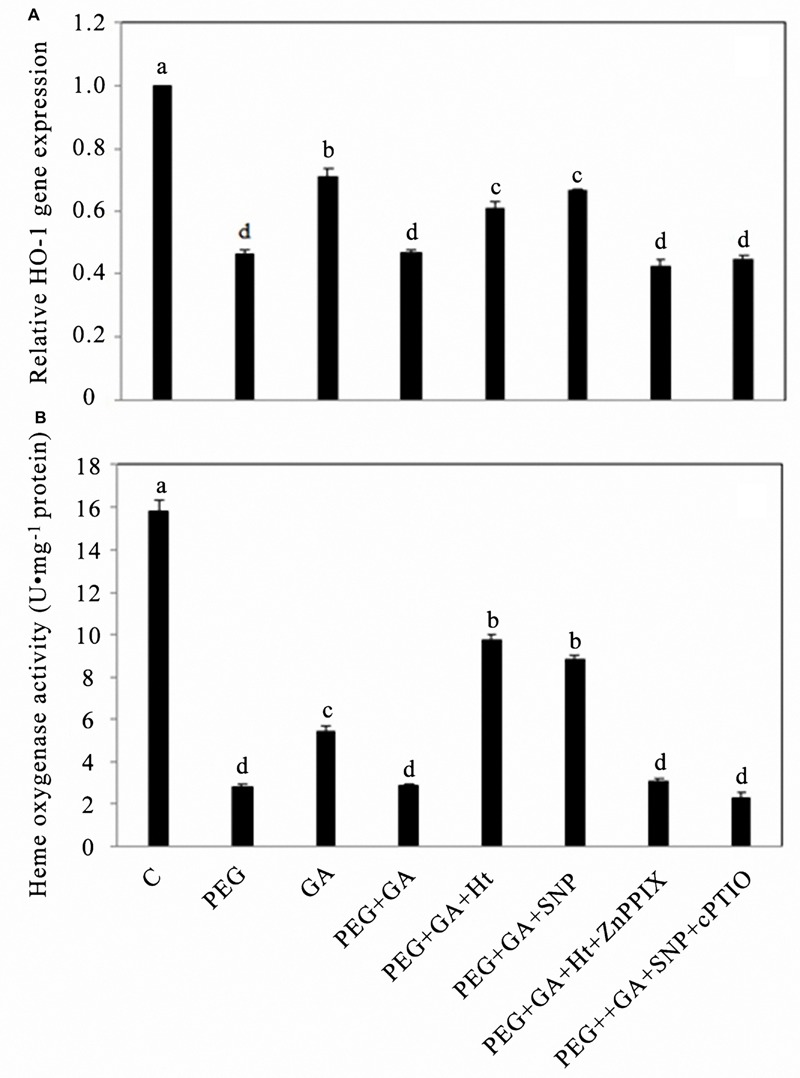
**Ht, SNP, ZnPPIX, and cPTIO up- or down-regulated the expression of *HO-1* and the activity of HO in GA-induced rice aleurone layers subjected to drought stress.** Aleurone layers treated with or without 20% PEG, 50 μM GA, 1 μM Ht, 200 μM SNP, 10 μM ZnPPIX, and 200 μM cPTIO alone or in combination for 24 h. A sample with distilled water treatment was used as control (C). The transcript levels of *HO-1* analyzed by real-time fluorescence quantitative PCR at 24 h. The expression levels of the *HO-1* gene are presented as values relative to that of the control **(A)**. Meanwhile, the corresponding HO activity **(B)** was determined after different treatments for 24 h. Mean values were calculated from at least three independent experiments, bars with different letters indicate significant differences at the 0.05 level, according to Duncan’s multiple test.

By fluorescent QRT-PCR relative analysis, we compared the results with those using distilled water (C, control), the PEG alone treatment significantly inhibited the expression level of *HO-1* by 44%, and exogenous GA treatment also inhibited the expression level of *HO-1* by 27%, but the treatment combined GA with PEG decreased by 33.80% compared to that observed in the GA alone treatment (**Figure 3A**). The treatments PEG + GA + Ht and PEG + GA + SNP partly eased the reduction in the *HO-1* expression level, compared to the treatment with PEG + GA, and respectively, increased by 29.79 and 42.55% (**Figure 3A**). The expression levels of the *HO-1* gene showed no significant differences among treatments PEG, PEG + GA, and PEG + GA in the presence of exogenous HO-1 inhibitor or NO scavenger, and changes in HO activity changes induced by these reagents were identical (**Figure 3B**). The HO-1 activities of rice aleurone layers treated with exogenous GA alone or PEG + GA were significantly inhibited (*P* < 0.05 or *P* < 0.01; **Figure 3B**). Both treatments PEG + GA + Ht and PEG + GA + SNP partly, respectively, raised the HO-1 activities inhibited by the treatment PEG + GA by 2.34- and 2.04-fold (**Figure 3B**). The treatment of HO-1 inhibitor, ZnPPIX, being added into PEG + GA + Ht, compared to the treatment PEG + GA + Ht, significantly reduced the HO-1 activity that was induced by Ht, thus indicating that the response was reserved by ZnPPIX (**Figure 3B**). Because of the participation of NO scavenger cPTIO, the treatment PEG + GA + SNP + cPTIO significantly decreased the effects of NO on raising the HO-1 activity of aleurone cells treated with PEG + GA (**Figure 3B**).

### GA, HO-1, and NO Promoted or Delayed PCD in Rice Aleurone Layers Under Drought Stress

The aims of the present study were to examine, whether HO-1 performs a crucial function in the induction of PCD in germinating rice aleurone layers and to confirm whether this effect is caused by its interaction with NO and GA signaling.

FDA and FM4-64 are fluorescence dyes commonly used to identify cell viability, wherein live cells emit green fluorescence and dead cells red fluorescence. This facilitates in distinguishing between live and dead cells in the aleurone layers.

**FIGURE 4:**
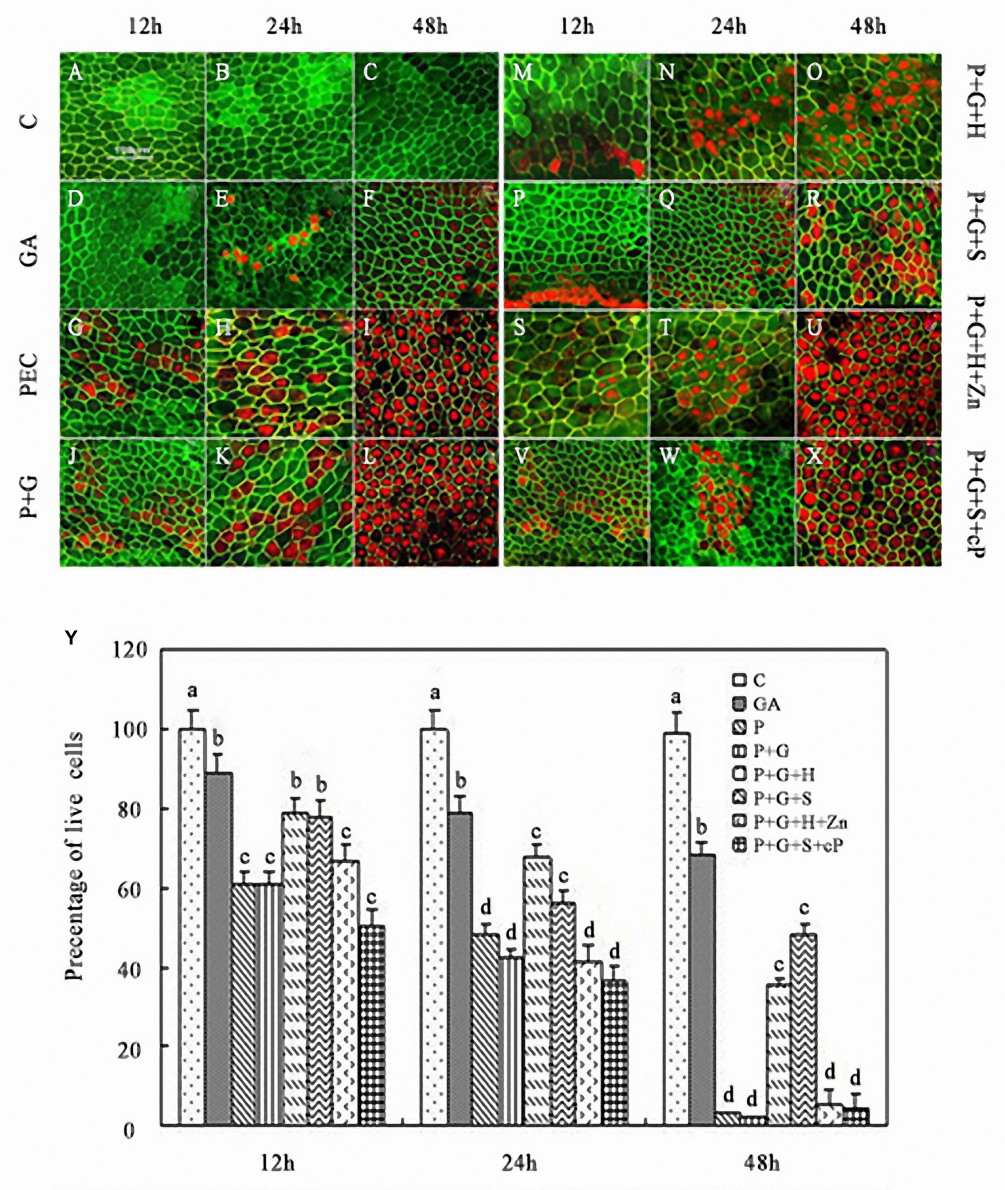
**Increase or decrease in *HO-1* gene expression and HO activity related to the acceleration or delay of GA-triggered PCD in rice aleurone layers under drought stress.** Aleurone layers were incubated in FDA (green, live cells) and FM-4-64 (red, dead cells) prior to image capture. Aleurone layers treated with or without 20% PEG (P), 50 μM GA (G), 1 μM Ht (H), 200 μM SNP (S), 10 μM ZnPPIX (Zn), and 200 μM cPTIO (cP) alone or in combination for 12, 24, and 48 h, respectively. A sample with distilled water treatment was used as control (C). Images of cells showing fluorescently labeled aleurone layers were captured **(A–X)**. Cell survival rate was also quantified **(Y)** at 12, 24, and 48 h. Scale bar, 100 μm. Mean values were calculated from at least three independent experiments, bars with different letters indicated significant differences at the 0.05 level, according to Duncan’s multiple test.

FDA and FM4-64 dual fluorescent staining combined with LSCM showed that the number of dead cells gradually prolonged the incubation time of rice aleurone layers subjected to various treatments (**Figures 4A–X**). Dead cells did not appear in rice aleurone layers within 24 h of distilled water culture (C, control), and most of cells showing green fluorescence after 48 h of incubation were still alive (**Figures 4A–C**). After treating aleurone layers with exogenous GA for 24 h, some cells died, and after 48 h, more than half of the cells were dead (**Figures 4D–F**). On the other hand, PEG and co-treatment with PEG and GA accelerated the death rate of aleurone cells; as early as 12 h after treatment, cells were dead, and within 48 h, almost all cells were dead in the aleurone layer (**Figures 4G–L**). While the treatments containing the HO-1 inducer or NO donor elevated the survival rate of the aleurone layer cells at 12, 24, and 48 h, even after 48 h, 35.67 and 48.33% of the cells remained alive (**Figures 4M–R**). On the other hand, treatments PEG + GA + Ht + ZnPPIX and PEG + GA + SNP + cPTIO showed no distinct differences at each time point, almost all cells of the aleurone layer were dead within 48 h of treatment (**Figures 4S–X**). These findings illustrated that the respective inhibitor and scavenger reversed the effects of HO-1 and NO on delaying PCD.

A coordinate curve of cell survival rate and time of various treatments was constructed (**Figure 4Y**). The aleurone layers showed a 90% cell survival rate within 48 h of distilled water treatment, whereas that treated with exogenous GA alone decreased to 45% after 24 h of drought stress, and almost no live cells were observed after 48 h. The cell survival rates of aleurone layers remained at 38 and 33% after 48 h of incubation in PEG + GA + Ht and PEG + GA + SNP, respectively, whereas when the treatments PEG + GA + Ht + ZnPPIX and PEG + GA + SNP + cPTIO were applied to the aleurone layer for 48 h, the cells remained alive (**Figure 4Y**), indicating that HO-1 and NO delayed the GA induction the cell death in rice aleurone layers subjected to drought stress.

### GA Accelerates the Development of a Large Central Vacuole in Aleurone Cells Under Drought Stress, and HO-1 and NO Slows the Effect of GA

The large central vacuole of aleurone cells is the prelude to PCD during cereal seed germination (Bethke et al., 1999). Therefore, our next goal was to explore, whether the vacuolation was associated with the PCD of aleurone layers in germinating rice seeds subjected to drought stress.

**FIGURE 5:**
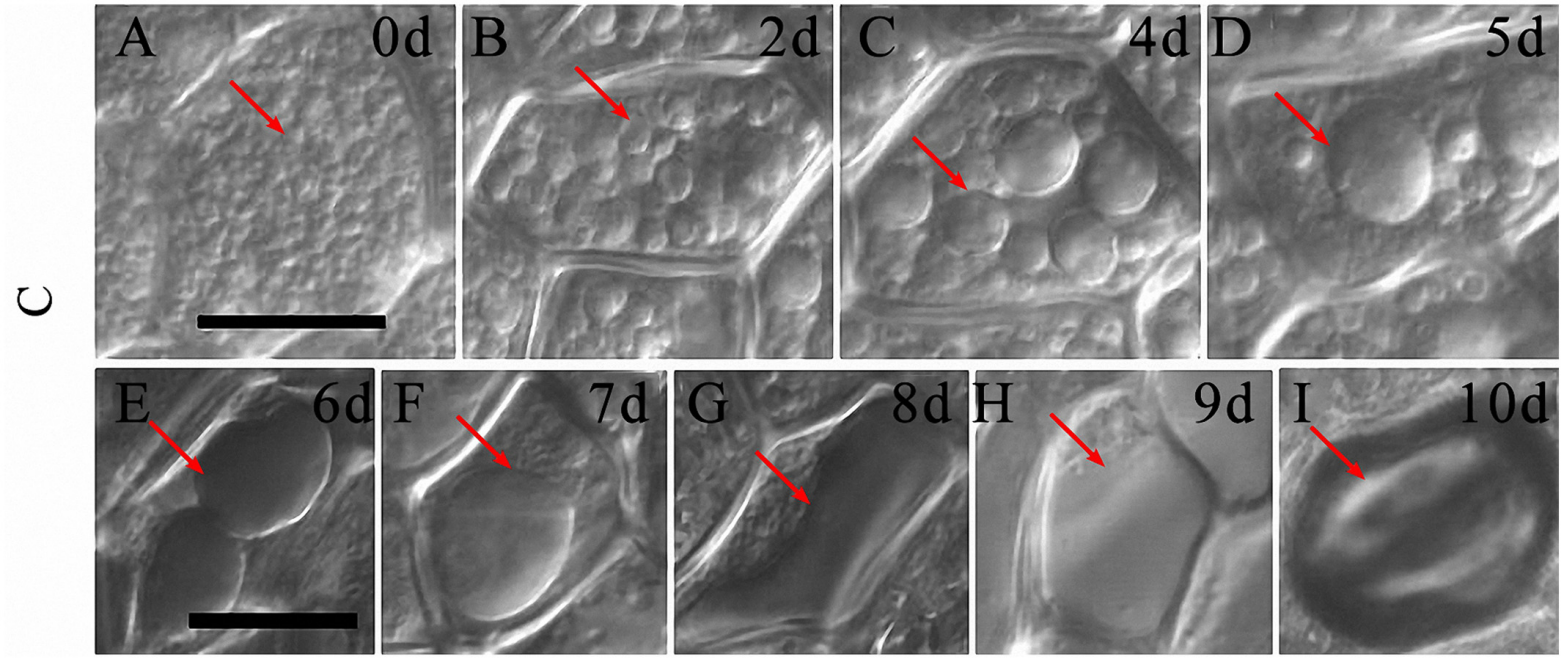
**The vacuolated process of aleurone cells treated with distilled water. (A–I)** Vacuoles of aleurone cells treated with distilled water at 0, 2, 4, 5, 6, 7, 8, 9, and 10 days. The scale is 20 μm.

By exploring morphological changes of aleurone cells during PCD under normal cultured conditions, we determined that the morphological changes of PSVs were significant in the aleurone cells. Therefore, based on the morphological changes in PSVs, the cell death process of rice aleurone layers could be determined. From 0 to 1 day of culture, aleurone cells were hexagonal, with nearly uniform wall thickness, and with spherical-shaped aleurone grains (**Figure 5A**, arrow). When aleurone layers were cultured from 2 to 6 days, the PSVs of aleurone cells began to merge into larger vesicles (**Figures 5B–E**, arrows) accompanied by vacuolation, a large central vacuole appeared after 7 days (**Figure 5F**, arrow), and then the large central vacuole deformed and elongated, and integrity of the vacuole membrane disappeared after 8 days (**Figure 5G**, arrow). Finally, vacuole rupture (**Figure 5H**, arrow) resulted in protoplast dissolution, followed by shrinking into a ball after 10 days (**Figure 5I**, arrow).

**FIGURE 6:**
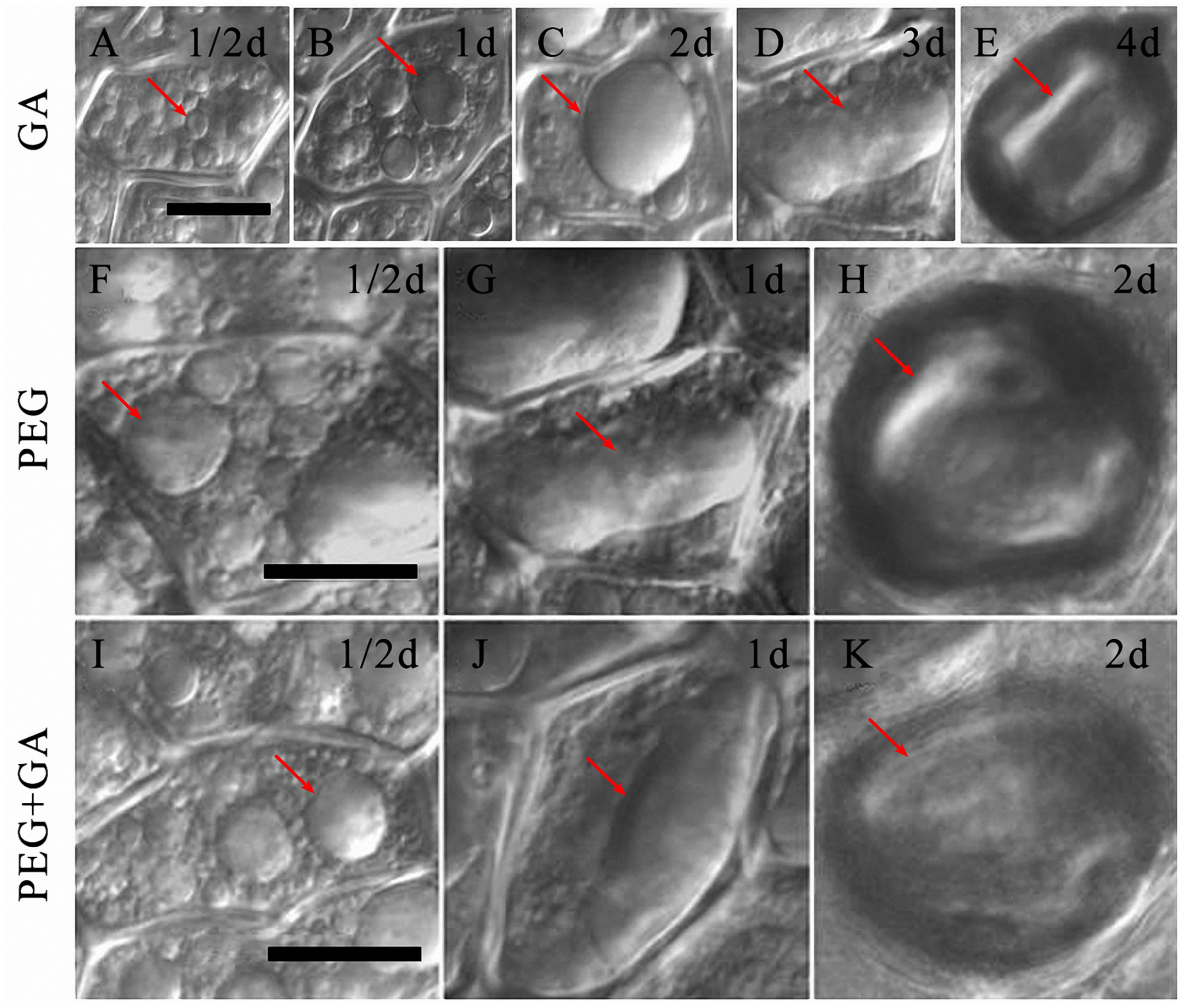
**Gibberellin (GA) co-treated with PEG accelerated the vacuolated process of aleurone cells. (A–E)** Vacuoles in aleurone cells treated with 50 μM GA at 1/2, 1, 2, 3, and 4 days. **(F–H)** Vacuoles in aleurone cells treated with 20% PEG for 1/2, 1, and 2 days. **(I–K)** Vacuoles in aleurone cells treated with 20% PEG + 50 μM GA for 1/2, 1, and 2 days. The scale is 20 μm.

In the GA alone treatment, several small PSVs were observed in the aleurone cells within 1/2 day (**Figure 6A**, arrow). However, the PSVs began to fuse and formed into several bigger vacuoles in GA-treated aleurone cells after incubation for 1 day (**Figure 6B**, arrow), and then these larger PSVs fused to form a large central vacuole after 2 days (**Figure 6C**, arrow). The large central vacuole elongated and deformed, and the deformed large central vacuole showed an unclear boundary after 3 days (**Figure 6D**, arrow), and finally, protoplasts appeared shrunken within cells after 4 days (**Figure 6E**, arrow). The death process of aleurone cells was earlier at 6 days compared to cells cultured under normal conditions. These results confirmed that exogenous GA significantly accelerates the vacuolation processes in aleurone cells.

Compared to the GA alone treatment, the PEG and PEG + GA treatments hastened the occurrence of the large central vacuole. The PSVs of aleurone cells treated with PEG and PEG + GA were larger than those treated with GA after 1/2 day (**Figures 6F,I**, arrows), exhibiting a large central vacuole with distinct deformation after 1 day (**Figures 6G,J**, arrows) and only after 2 days, presented the phenomenon of cell protoplasts mixing together and shrinking (**Figures 6H,K**, arrows). The results certified that drought stress speeds up the formation and rupture of large central vacuoles in GA-inducing aleurone cells.

**FIGURE 7:**
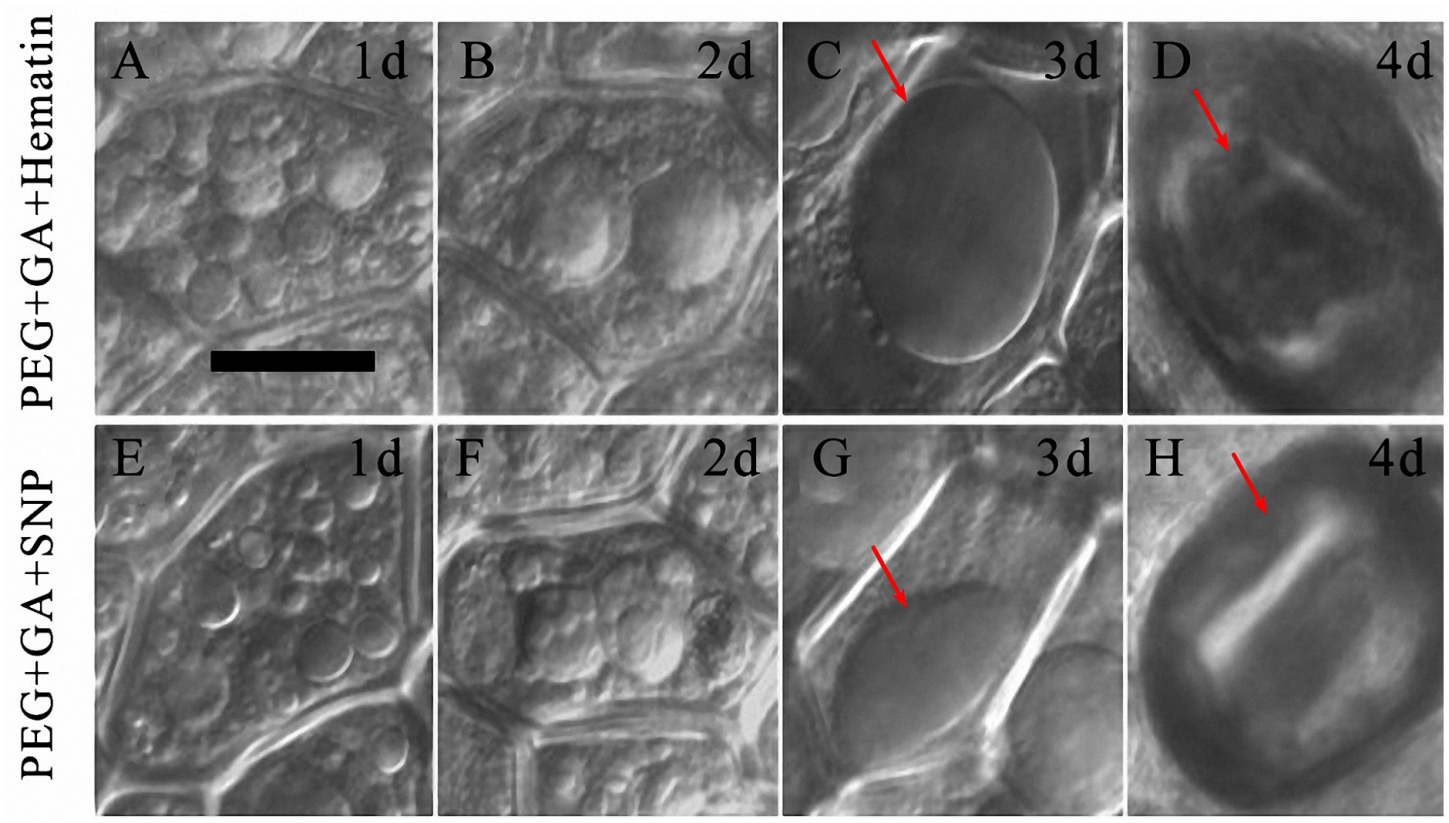
**Ht and SNP delayed vacuolation in aleurone cells treated with PEG plus GA. (A–D)** Vacuoles of aleurone cells treated with 20% PEG + 50 μM GA + 1 μM Ht at 1, 2, 3, and 4 days. **(E–H)** Vacuoles in aleurone cells treated with 20% PEG + 50 μM GA + 200 μM SNP for 1, 2, 3, and 4 days. The scale is 20 μm.

When the HO-1 inducer and NO donor were, respectively, added to PEG + GA, a large central vacuole of aleurone cell was not observed at 1 day (**Figures 7A,E**) and 2 days (**Figures 7B,F**). A large central vacuole did not emerge until 3 days of the PEG + GA + Ht and PEG + GA + SNP treatments (**Figures 7C,G**, arrows), cell protoplast shrinking was only observed after 4 days (**Figures 7D,H**, arrows), which was then followed by cell death. The occurrence of large central vacuoles was observed two days later in both PEG + GA + Ht and PEG + GA + SNP treatments compared to that in the PEG + GA treatment (**Figures 6I–K**). These results suggest that HO-1 and NO delayed the occurrence of large central vacuoles in GA-induced aleurone cells subjected to drought stress. Therefore, HO-1 and NO contribute to the delay in GA-induced aleurone layers PCD during drought stress.

## Discussion

In our previous study, HO-1 inducer Ht differently up-regulated the activities of SOD, CAT, and peroxidase (POD), resulting in the apparent decrease of membrane permeability and malondialdehyde (MDA) content. These results suggested that HO-1 alleviates drought-induced oxidative damage in rice seeds germination (data not shown). Increasing evidence demonstrated that endogenous ROS, particularly H_2_O_2_, play a critical role in regulating the PCD of cereal aleurone layers (Bethke and Jones, 2001; Wu et al., 2011). Antioxidants and ROS scavengers such as butylated hydroxytoluene (BHT), dithiothreitol (DTT), and ascorbic acid (AsA) not only delayed PCD, but also mimicked the effect of HO-1 inducer haematin on up-regulating HO-1. In addition, haematin blocked the decrease of GA-induced ascorbate peroxidase (APX) and CAT activities, and then resulted in the decrease of H_2_O_2_ level, and these effects were reversed by HO-1 inhibitor ZnPPIX (Wu et al., 2011). The up-regulation of HO-1 would contribute to the capability of HO, act as a potent antioxidant enzyme, eliminating ROS and protecting cells from oxidative stress in wheat aleurone layers (Wu et al., 2011, 2014), which has previously been confirmed in plants (Noriega et al., 2004; Han et al., 2008). The present study showed that the up-regulation of HO contributed to the delay in PCD in rice aleurone layers induced by GA under drought stress, the similar results were obtained by Wu et al. (2011, 2014). Above mentioned results revealed that HO-1 function in the PCD of cereal aleurone layers is dependent on enhancing the activities of antioxidant enzymes.

Mounting evidence has confirmed that HO-1 and NO play important physiological functions in plants, such as improving resistance and promoting seed germination. After treatment with Ht, the gene transcription and activity level of HO-1 were up-regulated, as well as alleviated Cd-induced oxidative damage in alfalfa root tissue (Han et al., 2008). NO, as an antioxidant, reduced the accumulation of ROS, and alleviated the damage caused by oxidative stress, thereby enhancing the adaptability of plants to abiotic stress (He et al., 2014). In addition, exogenous NO donor SNP induced the up-regulation of HO-1 (Noriega et al., 2007; Xuan et al., 2008; Santa-Cruz et al., 2010), and NO prolonged the life of barley aleurone cells treated with GA (Beligni et al., 2002). The aleurone layers of cereal grains undergo PCD, which is regulated by GA following germination (Domínguez et al., 2004). By exploring the relationship between HO-1 and NO during PCD in rice aleurone layers, we discovered that the HO-1 inducer Ht and NO donor SNP up-regulated the gene expression and activity of HO-1 in the rice aleurone layers under drought stress, but their effects were, respectively, reversed by specific inhibitor ZnPPIX and scavenger cPTIO. Simultaneously, the effect of HO-1 was blocked by NO scavenger cPTIO, whereas HO-1 activity inhibitor ZnPPIX did not block the effect of NO on the up-regulation of *HO-1* gene expression and activity of aleurone layers (**Figures 1A,B**). In addition, treatments using HO-1 inducer or NO donor resulted in an increase in endogenous NO content in aleurone cells under drought stress (**Figures 2A,B**). Similar results were observed using SNP and Ht, which accelerated NO emission in etiolated wheat seedling leaves (Liu et al., 2013). Further experiments showed that the NO scavenger repressed the action of the HO-1 inducer Ht and NO donor SNP. However, the HO-1 inhibitor ZnPPIX was unable to block the effect of the NO donor of increasing the level of endogenous NO (**Figures 2A,B**). Furthermore, compared to the Ht alone-treated sample, ZnPPIX and cPTIO markedly reduced the Ht-induced up-regulated NO level, suggesting that HO-1-mediated NO production is a major source of endogenous NO in aleurone layers. Based on these results, we deduced that there is a link between HO-1 and NO in GA-induced PCD in rice aleurone layers subjected to drought stress. Similarly, SNP- and Ht-triggered up-regulation of HO-1 was observed in soybean seedlings (Noriega et al., 2007; Santa-Cruz et al., 2010) and wheat aleurone layers (Wu et al., 2011). Li et al. (2015) inferred that HO-1 acting downstream of NO signaling was involved in β-CDH-induced lateral root formation in tomato. A different conclusion was also drawn in that NO acted downstream of HO-1, wherein it is involved in hemin-induced cucumber adventitious rooting (Xuan et al., 2012). Another study also showed that HO stimulated rooting of mung bean hypocotyl cuttings upstream of NOS/NO (Xu et al., 2006).

The aims of the present study were to examine whether HO-1 performs a crucial function in responding to the GA-induced PCD of germinating rice aleurone layers and to confirm whether this effect is caused by its interaction with NO. Previous studies have confirmed that GA initiated PCD in the aleurone layers of wheat (Kuo et al., 1996) and barley (Wang et al., 1996; Bethke et al., 1999). In the present study, GA and PEG, and PEG co-treated with GA significantly reduced the gene expression and activity of HO-1 (**Figures 3A,B**), thereby accelerating PCD in aleurone layers (**Figures 4D–L**), whereas the HO-1 inducer Ht and NO donor SNP alleviated the GA-induced decline in *HO-1* gene expression and activity in rice aleurone layers subjected to drought stress (**Figures 3A,B**), improved cell survival rate, and delayed the occurrence of GA-induced aleurone layer PCD in the germinating rice seeds under drought stress (**Figures 4M–R**). The effects of the HO-1 inducer and NO donor SNP were, respectively, reversed by the HO-1 inhibitor ZnPPIX and NO-specific scavenger cPTIO, whereas PCD in rice aleurone layers was accelerated (**Figures 4S–X**). A previous study suggested that the HO inducer haematin increases HO transcription and activity, and inhibits PCD of wheat aleurone layers, whereas the HO inhibitor ZnPPIX reverses the effect of haematin and contributes to cell death (Wu et al., 2011). These results ensured that intracellular HO plays a major role in postponing the PCD of aleurone layers subjected to drought stress.

The vacuole plays a crucial role in the PCD of plants (Gucciardi et al., 2004; Gadjev et al., 2008; Xiao et al., 2009; Hara-Nishimura and Hatsugai, 2011), wherein only highly vacuolated cells undergo PCD in plants (Bethke and Jones, 2001). Therefore, we wanted to know, whether the degree of vacuolation is associated with PCD in rice aleurone layers. In the present study, we determined that variation in vacuole morphology of aleurone cells sequentially occurred under normal culture conditions; it took 7 days for the large central vacuole to emerge (**Figures 5A–F**). Subsequently, the large central vacuole lost its distinct boundary and in turn underwent deformation, thereby resulting in tonoplast rupture (**Figures 5G–I**). However, GA increased the rate of vacuolation of PEG-induced aleurone layers (**Figures 6I–K**). Therefore, a large number of cells in the aleurone layers were dead (**Figure 4L**). Interestingly, HO-1 inhibitor ZnPPIX and NO scavenger cPTIO mimicked the effect of GA-promoted vacuolation and PCD, and HO-1 inducer Ht and NO donor SNP delayed the appearance of large central vacuoles (**Figures 7C,G**), ultimately resulting in the delay in the onset of PCD in rice aleurone layers subjected to drought stress. Therefore, we speculated that HO-1 and NO are involved in postponing GA-induced vacuolation of aleurone cells in rice under drought stress by preventing the formation of large central vacuoles.

In the present study, we showed that up-regulating HO alleviates GA-induced PCD in rice aleurone layers subjected to drought stress. A similar finding involving the up-regulation of HO that results in a delay in PCD in wheat has been earlier described (Wu et al., 2011).

Previous studies have confirmed that vacuoles are essential in initiating PCD in plants, and vacuole membrane rupture is a critical step in plant PCD (Higaki et al., 2011). However, previous studies did not determine, whether changes in *HO-1* gene expression affected the process of vacuolation and PCD. Therefore, we investigated the relationship of up- or down-regulation of HO-1, vacuolation, and GA-induced PCD progression in rice aleurone layers subjected to drought stress. The results indicated that GA, the HO-1 inhibitor, and the NO scavenger down-regulate the *HO-1* mRNA level and HO activity, which in turn increases the rate of vacuolation and PCD. However, the HO-1 inducer and NO donor slowed down the process of vacuolation and PCD by up-regulating *HO-1* gene expression and HO activity.

In summary, we present evidence that suggest that GA regulates the expression of HO-1 in germinating rice aleurone layers subjected to drought stress. The level of *HO-1* gene expression and HO activity plays an important role in ascertaining the process of GA-induced PCD in response to drought stress. HO-1 and NO modulate each others function in aleurone layers, similar to that in the NO donor SNP, wherein Ht-driven HO-1 promotes the level of endogenous NO. Correspondingly, the enhanced NO triggers the up-regulated expression and activity of HO-1, whereas the NO inhibitor cPTIO down-regulated the expression and activity of HO-1. Therefore, the observed mutual induction effects indicate that there might be an inseparable relationship between HO-1 and NO in delaying the PCD of GA-induced rice aleurone layers subjected to drought stress. However, the PCD of cereal aleurone layers is a complex event, and it is unable to be elucidated clearly only depending on the evidence of biochemistry, cell morphology, and pharmacology. Therefore, future studies aim to investigate the HO, NO, and GA signal transduction pathways and the molecular mechanism in the PCD of rice aleurone layers subjected to drought stress by combining with molecular methods.

## Author Contributions

HC designed the experiment. HC and HW wrote the paper. HW carried out fluorescence quantitative RT-PCR. YZ carried out cell morphology observation and subcellular localization. JL helped in enzyme activity determination. HC and HZ helped in drafting the manuscript.

## Conflict of Interest Statement

The authors declare that the research was conducted in the absence of any commercial or financial relationships that could be construed as a potential conflict of interest.
